# BioModels’ model of the year 2024

**DOI:** 10.3389/fsysb.2026.1829848

**Published:** 2026-07-08

**Authors:** Rahuman S. Malik Sheriff, Hiroki Asari, Liesbet Geris, Henning Hermjakob, Karthik Raman, Blerta Shtylla, Maria Zimmermann-Kogadeeva

**Affiliations:** 1 European Bioinformatics Institute (EMBL-EBI), European Molecular Biology Laboratory, Hinxton, Cambridge, United Kingdom; 2 Department of Surgery and Cancer, Imperial College London, London, United Kingdom; 3 Laboratory for Systems Medicine, Department of Medicine, University of Florida, Gainesville, FL, United States; 4 Epigenetics and Neurobiology Unit, European Molecular Biology Laboratory (EMBL), Monterotondo, Italy; 5 KU Leuven, Department of Mechanical Engineering (Biomechanics Section), Leuven, Belgium; and University of Liège, GIGA In Silico Medicine, Liège, Belgium; 6 Department of Biotechnology and Robert Bosch Centre for Data Science & AI (RBCDSAI), Indian Institute of Technology Madras, Chennai, India; 7 Pharmacometrics & Systems Pharmacology, Translational Clinical Sciences, Pfizer R & D, La Jolla, San Diego, CA, United States; 8 Genome Biology Unit, European Molecular Biology Laboratory (EMBL), Heidelberg, Germany

**Keywords:** cardiac electrophysiology, intestinal crypt dynamics, MASLD signalling, model reproducibility, post-infarction electromechanics, systems biology modelling

## Abstract

Computational modelling is a widely used approach for understanding complex biological systems, enabling researchers to formalise mechanisms, analyse dynamic behaviours, and generate predictive insights across multiple biological scales. The BioModels *Model of the Year (MOY) 2024* competition was organised to recognise outstanding modelling contributions from the systems biology community, with a particular focus on early-career researchers and models that demonstrate strong scientific impact, technical rigour, and reproducibility. The initiative also promotes best practices in model dissemination by encouraging submissions that follow community standards and adhere to FAIR principles. Here, we describe the MOY2024 selection process and present the four winning models curated in BioModels. The award-winning submissions span diverse areas of biomedical research, including hepatocyte signalling in metabolic liver disease, cardiac electrophysiology for drug safety assessment, multiscale modelling of intestinal epithelial dynamics, and electromechanical modelling of post-infarction cardiomyocytes. Together, these reproducible and reusable systems biology models illustrate how computational frameworks can advance both fundamental biology and translational biomedical research.

## 1 Introduction

Computational models are indispensable tools for investigating complex biological systems. They allow researchers to formalise mechanisms into mathematical formulations such as ordinary differential equations (ODEs), stochastic differential equations, agent-based models, logic models, and genome-scale metabolic models. Each of these approaches offers distinct strengths, enabling analyses that span molecular interactions, cellular behaviour, and even whole-organism physiology.

Despite their strong mathematical foundations, reproducibility challenges persist. A systematic evaluation of 455 published models revealed that nearly half of them could not be reproduced with the information provided in the manuscript, often due to incomplete reporting or errors in implementation, prompting the introduction of an eight-point reproducibility scorecard to guide higher standards for authors, reviewers, and editors (Tiwari et al., 2021).

Over the past 2 decades, **BioModels** has played a central role in addressing these challenges and advancing open science. As the world’s largest repository of curated systems biology models, it ensures that models are encoded in standardised formats, annotated with controlled vocabularies, and shared in accordance with FAIR (Findable, Accessible, Interoperable, Reusable) principles. Authors can submit their models in any modelling approaches as long as they are biologically relevant. These models can be submitted in any format or programming language, however community standards such as SBML or CellML are recommended when plausible. During the curation process, an expert curator ensures the model code accurately reflects the equations and parameters in the original manuscript, updates the code if necessary and reproduces at least one published simulation figure in the article. Reproducible models are annotated with controlled vocabularies and published under the “curated models” branch in BioModels (Malik-Sheriff et al., 2020). BioModels not only archives published models but also adds value by ensuring reproducibility, thereby accelerating reuse in new studies, training, and industrial applications.

To celebrate excellence in computational modelling, BioModels launched the **Model of the Year (MOY)** competition in 2023 (Malik Sheriff et al., 2024). Applications were specifically invited from early career researchers across academia and industry, providing a platform to showcase their work and recognise their contributions. The initiative highlighted models of high scientific merit, reproducibility, and adherence to FAIR principles. Building on this foundation, the **MOY2024** competition once again identified key models from the systems biology community and showcased the creativity, rigour, and broader impact of modelling in both fundamental research and applied contexts.

## 2 Selection process

The MOY2024 competition attracted 30 eligible submissions, spanning a broad spectrum of biological systems and modelling approaches. Submissions ranged from fundamental mechanistic models to those with direct industrial applications.

Each model was independently assessed by an expert jury according to defined criteria: scientific merit, technical rigour, novelty, biological relevance, and validation against experimental data. The jurors’ evaluations were guided by the question of whether the model deserved recognition as the Model of the Year 2024, with responses captured on a five-point scale (Definitely Yes, Yes, Maybe, No, Abstain).

**FIGURE 1:**
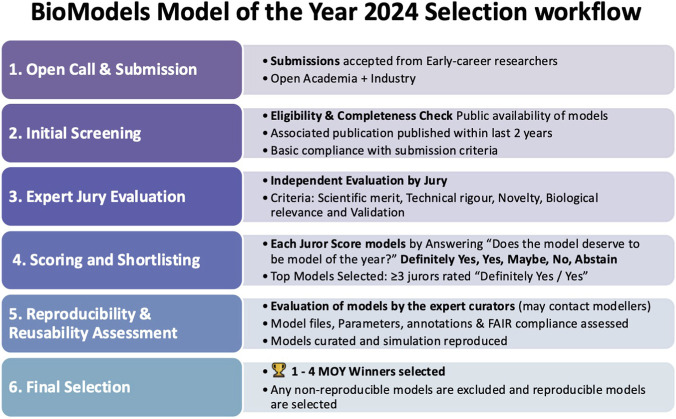
The selection workflow for the BioModels Model of the Year 2024.

The assessment showed mixed views; no model received unanimous top-tier support from all jurors. Models with at least three out of five jurors rating it “Definitely Yes” or “Yes” were subsequently subjected to a deeper review of their reproducibility and reusability by independent model curators, reflecting BioModels’ mission to promote models that are not only scientifically strong but also FAIR and reproducible. From this two-stage evaluation, four winners were selected as the MOY2024 awardees. The steps involved in the model selection are shown in detail in Figure 1. Each winning model exemplifies excellence in combining biological insight with robust methodology, providing valuable resources for the community.

## 3 MOY2024 winners

- **Svenja Kemmer**–*Hepatocyte Growth Factor Signaling in Fatty Liver Disease*
  Advisor: Jens Timmer, Institute of Physics, University of Freiburg, Germany
  BioModels ID: MODEL2306280002 (Burbano De Lara et al., 2024)
- **Marcel Mohr**–*Rabbit Purkinje Cardiac Electrophysiology*
  Advisor: Friedemann Schmidt, Sanofi, Frankfurt/Main, Germany
  BioModels ID: MODEL2204250001 (Mohr et al., 2022)
- **Xin Zhou, Jakub Tomek, Zhinuo Jenny Wang**–*Post Infarction Myocyte Electromechanics*
  Advisor: Blanca Rodriguez, University of Oxford, United Kingdom
  BioModels ID: MODEL2402290004 (Zhou et al., 2024)
- **Louis Gall**–*Agent-based Model of the Intestinal Epithelium*
  Advisor: Carmen Pin, AstraZeneca, Cambridge, United Kingdom
  BioModels ID: MODEL2212120002 (Gall et al., 2023).

## 4 Hepatocyte Growth Factor Signaling in Fatty Liver Disease

This model addresses a central biological question: how nutrient-rich Western diets alter hepatocyte signal transduction and contribute to metabolic dysfunction-associated steatotic liver disease (MASLD), a disorder with rising prevalence and risk of progression to cancer. The model specifically investigates Hepatocyte Growth Factor (HGF) signalling, focusing on the role of basal phosphorylation of the MET receptor as an indicator of hepatocyte dysregulation.

Developed with coupled ODEs, the model integrates MET receptor activation, nutrient-sensitive mTOR signalling, and downstream MAPK and PI3K/AKT cascades, the key regulators of hepatocyte proliferation and regeneration. It includes 23 molecular species and 26 reactions, calibrated using quantitative, time- and dose-resolved immunoblotting data from mouse hepatocytes under standard and Western diet conditions, together with proteomics data to constrain basal protein abundances.

Simulations revealed that a single parameter—the basal phosphorylation rate of MET—could account for the major signalling differences observed between healthy and steatotic hepatocytes, including reduced AKT activation and enhanced growth factor-independent proliferation. Extending the model to human hepatocytes, calibration showed that patient-specific basal MET phosphorylation correlated with post-operative recovery, positioning it as a predictive biomarker for liver health.

By linking dietary impact, molecular signalling, and clinical outcome, this model provided a mechanistic explanation for MASLD progression and offered translational value. This model exemplifies how dynamic modelling can support both fundamental systems biology and clinical decision-making in liver disease (Burbano De Lara et al., 2024).

## 5 Rabbit Purkinje Cardiac Electrophysiology

This model addresses the key challenge of predicting drug-induced arrhythmias, one of the major causes of drug attrition. The biological question was whether a simplified electrophysiology model of rabbit Purkinje fibres could reliably reproduce experimental findings from the rabbit Purkinje fibre assay, a gold-standard test in safety pharmacology.

Built on a Hodgkin–Huxley formulation, the model incorporates the most important ionic currents responsible for action potential (AP) genesis, while deliberately omitting intracellular ion concentration dynamics to reduce complexity and computational cost. Drug effects are simulated using a conductance block formalism, which reduces the maximal conductance of specific ion channels based on their inhibition constants (IC50) and drug concentrations.

Calibration and benchmarking were performed against an extensive dataset of approximately 588 Purkinje fibre experiments covering about 560 compounds with diverse mechanisms of action. The tailored model quantitatively reproduced 80% of experimental outcomes, outperforming more complex models in accuracy while requiring far less computational effort.

These results demonstrate that basal AP morphology and drug-induced changes in AP duration can be robustly captured by a minimalistic model. Curated in CellML format in BioModels, it provides a validated, reproducible tool for lead optimisation and cardiac risk assessment, offering significant benefits for pharmaceutical research by reducing reliance on animal testing and enabling efficient early hazard identification (Mohr et al., 2022).

## 6 Agent-based model of the intestinal epithelium

This model addresses the biological question of how the intestinal epithelium maintains homeostasis and responds to injury or drug-induced toxicity. It was developed as a multiscale agent-based model (ABM) of the mouse intestinal crypt, where each simulated cell interacts physically and biochemically, undergoes cell cycle progression, and differentiates into specialised epithelial lineages. The ABM incorporates multiple regulatory pathways such as Wnt, Notch, BMP, ZNRF3/RNF43, and Hippo-YAP, that control proliferation, differentiation, and tissue organisation, while cell cycle progression is described using a mechanistic model with DNA/RNA integrity checkpoints.

The ABM was calibrated to reproduce the stable crypt structure observed experimentally, maintaining a balance between proliferating, differentiating, and migrating cells. Simulations examined three injury scenarios: targeted stem cell ablation, CDK1 inhibition, and 5-fluorouracil (5-FU)–induced DNA/RNA damage. The model recapitulated experimental observations, showing crypt regeneration via dedifferentiation of mature cells after stem cell loss, and extensive epithelial disruption when drug treatments impaired cell cycle progression, leading to apoptosis, reduced proliferation, and eventual loss of barrier integrity.

These results demonstrate how perturbations at the intracellular level propagate to tissue-scale consequences. With its modular and expandable design, the ABM provides a platform to integrate molecular and cellular data into predictions of organ-level outcomes. This model serves as a valuable tool for fundamental studies of epithelial biology and for industrial applications in predictive toxicology and drug development (Gall et al., 2023).

## 7 Post Infarction Myocyte Electromechanics

This model addresses the clinical problem of risk stratification after myocardial infarction, where patients face variable phenotypes and unpredictable arrhythmic risk. The central question was whether electromechanical modelling could disentangle the relationship between post-infarction electrical remodelling, contractile performance, and arrhythmia susceptibility.

The baseline model of healthy human ventricular myocytes was constructed with systems of ODEs and Markov chain formulations, integrating sarcolemmal ionic currents, calcium handling, and excitation–contraction coupling of the contractile apparatus. It was extensively validated against human experimental data, reproducing action potential morphology, calcium transients, active tension, rate dependence, and drug responses.

From this validated baseline, the authors developed multiple post-infarction variants to reflect acute and chronic remodelling stages. Three acute border zone models and three chronic border and remote zone models were created, each incorporating ionic current remodelling informed by experimental studies. At the cellular level, simulations produced heterogeneous action potential prolongations independent of calcium transient amplitude or contractile force. At the organ level, embedding these cell variants into ventricular simulations reproduced diverse ECG phenotypes observed clinically—such as T-wave inversion, Brugada-like patterns, and recovery of upright T-waves. Crucially, simulated ejection fraction did not correlate with electrophysiological heterogeneity, highlighting its inadequacy as a sole biomarker of arrhythmic risk.

By providing mechanistic evidence that contractile function and arrhythmia susceptibility can be dissociated, the model underscores the need for multifactorial patient risk assessment. This model serves as a reference for both fundamental cardiac electrophysiology research and clinical translation (Zhou et al., 2024).

## 8 Discussion

The MOY2024 winners exemplify the diversity and strength of computational systems biology, spanning molecular signalling, cardiac safety, tissue dynamics, and clinical cardiology. Across the MOY2024 winners, several common characteristics emerge, including clear alignment between the biological question and modelling approach, rigorous validation against experimental or clinical data, and adherence to community standards when possible, enabling FAIR and reproducible model dissemination.

The Reproducibility Scorecard provides simplified guidelines to support modellers (Tiwari et al., 2021). Recent community efforts have extended FAIR principles towards CURE (Complete, Understandable, Reproducible, and Executable) modelling, which emphasises that models should be fully specified, well-documented, and directly executable without ambiguity (Sauro et al., 2026). When models combine scientific novelty with FAIR and CURE principles, they become invaluable resources for the community. By providing comprehensive annotations, and complying with community standards, models are much more amenable for reuse, in various applications, from hypothesis testing to clinical translation.

Reproducibility continues to be a cornerstone of the field. Community-driven frameworks such as **FROG** for genome-scale metabolic models (Raman et al., 2024) and **EFECT** for stochastic models (Sego et al., 2024) are emerging to automate technical validation of simulation results. Together with BioModels’ established curation pipeline, these advances ensure that high-quality models remain reusable and integrable into future workflows.

Notably, the four winning models highlight how computational approaches cover multiple scales of biology, from molecular signalling (liver disease) and cellular electrophysiology (rabbit Purkinje fibres) to more complex tissue/organ-level dynamics (intestinal epithelium/heart myocyte electromechanics). Showcasing a rich variety of frameworks (ODEs, Hodgkin-Huxley models and agent-based modelling), all the models serve as excellent exemplars of mathematical modelling, illustrating how simplifying assumptions, when carefully calibrated and validated, can yield robust insights with both fundamental and translational value. Furthermore, each model tackles important biomedical challenges, ranging from metabolic disease to cardiac arrhythmias. The models’ focus on clinical outcomes reiterates the evolving role of computational models as not mere theoretical tools, but as important frameworks that can enhance confidence in translational outcomes.

The MOY initiative not only rewards scientific excellence but also the use of community standards, thereby motivating researchers to adopt best practices in FAIR and reproducible model dissemination. By celebrating the MOY2024 winners, we recognise the community’s progress towards reproducible, reusable, and impactful systems biology modelling. These models will serve as references for future work, inspiring the next-generation of tools, applications, and discoveries.

## Data Availability

The codes of models presented in the article are publicly available in the BioModels repository; further inquiries can be directed to the corresponding author.
